# Chemical Design and Therapeutic Efficacy of HBsAg-Based Nanovaccines for Hepatitis B Virus Clearance: A Preclinical and Clinical Systematic Review

**DOI:** 10.34172/apb.45985

**Published:** 2026-05-19

**Authors:** Hasti Nequi Marnani, Tahereh Zadeh Mehrizi, Babak Eshrati, Mehdi Shafiee Ardestani, Nazanin Ajdary, Mohammad Vodjgani, Nariman Mosaffa

**Affiliations:** ^1^Department of Radiopharmacy, Faculty of Pharmacy, Tehran University of Medical Sciences, Tehran, Iran; ^2^Health Management and Economics Research Center, Health Management Research Institute, Iran University of Medical Sciences, Tehran, Iran; ^3^Department of Community and Family Medicine, Faculty of Medicine, Iran University of Medical Sciences, Tehran, Iran; ^4^Research Center for Nuclear Medicine, Shariati Hospital, North Kargar Ave. 1411713135, Tehran, Iran; ^5^School of Pharmacy, International Campus, Tehran University of Medical Sciences, Tehran, Iran; ^6^Department of Immunology, School of Medicine, Tehran University of Medical Sciences, Tehran, Iran; ^7^Department of Immunology, School of Medicine, Shahid Beheshti University of Medical Sciences, Tehran, Iran

**Keywords:** Hepatitis B, Chronic, Nanovaccines, Hepatitis B surface antigens, Therapeutic

## Abstract

More than 240 million people worldwide are chronically infected with hepatitis B. Although current antiviral therapies effectively inhibit viral replication, they rarely result in a functional cure. The development of hepatitis B surface antigen (HBsAg)-based nanovaccines has emerged as a potential therapeutic strategy. The aim of this systematic review was to investigate a variety of formulations based on this system and determine their therapeutic efficacy in increasing antibody production, reducing HBsAg and hepatitis B virus DNA levels, stimulating T-cell responses, and reducing hepatitis B nuclear antigen (HBcAg)-positive hepatocytes. Studies published from 2015 to 2025 were included if they assessed the therapeutic benefits of HBsAg-based nanovaccines in animal models of chronic hepatitis B (CHB) or in human patients. A search of the PubMed/Medline, Scopus, and Web of Science databases yielded 361 papers, of which eight, including seven preclinical trials and one clinical trial, satisfied the inclusion criteria. Both HBsAg-containing protein-based and mRNA-based nanovaccines demonstrated a significant ability to increase antibody production, reduce viral markers (HBsAg, HBV DNA, HBcAg, and cccDNA), and induce robust T-cell activation, while maintaining liver enzyme levels (ALT and AST) within the normal range. PreS1-targeting platforms (such as Ferritin NP-preS1) and mRNA-based lipid nanoparticles encoding HBsAg demonstrated enhanced therapeutic efficacy. The well-tolerated εPA-44 liposome vaccines, which were in phase II trials, demonstrated favorable clearance of HBV DNA. In conclusion, HBsAg-based nanovaccines show promising therapeutic potential for CHB by restoring antiviral immunity and reducing viral persistence. Large-scale clinical trials are necessary to confirm their efficacy and long-term safety.

## Introduction

 Hepatitis B virus (HBV) is an enveloped DNA virus that selectively infects human hepatocytes to cause acute and chronic liver disease.^1,2^ Mother-to-child transmission is predominant in high-endemic areas, whereas sexual and parenteral routes have a greater contribution in low-endemic areas.^3^ Hepatitis B is often acquired at birth or in early life, leading to chronic HBV antigen exposure and immune system depletion.^4^ The infection results in abnormal T-cell function, like impaired cytotoxicity, defective cytokine production, and enhanced inhibitory receptors like PD-1 and cytotoxic T lymphocyte (CTL)-4.^4-6^ Most adults can clear an acute HBV infection by inducing specific CD4 ^+^ and CD8 ^+^ T cells and neutralizing antibodies, but infants and some adults cannot clear the infection and end up developing chronic infection and its complications instead.^6-9^ Chronic hepatitis B (CHB) is a serious infection caused by HBV, which is a sustained public health crisis affecting over 240 million individuals worldwide. WHO hopes to eliminate CHB by 2030 due to its liver morbidity and mortality burden.^10^ CHB can lead to cirrhosis, hepatocellular carcinoma (HCC), and liver failure, which account for approximately 30% of cirrhosis and 53% of HCC cases globally.^11,12^

 When a person gets infected, HBV relaxed circular DNA (rcDNA) is copied, moved into the nucleus, and cut by the body’s enzymes into covalently closed circular DNA (cccDNA), which serves as a template for ongoing viral replication. When a person gets infected, HBV relaxed circular DNA (rcDNA) is copied, moved into the nucleus, and cut by the body’s enzymes into covalently closed circular DNA (cccDNA), which serves as a template for ongoing viral replication.^13,14^ HBV genome contains three major surface antigens—small (S), middle (M), and large (L) proteins—collectively known as hepatitis B surface antigens (HBsAg).^15^ These are engaged in crucial viral replication and immune evasion processes. The S protein, or HBsAg, is the superior antigen for prophylactic HBV vaccines that have reduced HBV prevalence globally by creating protective immunity among immunocompetent hosts.^16^ These vaccines, however, do not produce sufficient hepatitis B surface antibodies (anti-HBs) in CHB patients due to chronic immune tolerance resulting from the elevated circulating levels of HBsAg.^17^ Immune deficiency is characterized by dysfunctional antigen-presenting cells (APCs), such as dendritic cells (DCs), and dysfunctional natural killer (NK) cells, B cells, and T cells, which is a major barrier to the therapeutic vaccine effectiveness.^18,19^

 Currently, treatments for CHB are mostly represented by two drug classes: interferon-α (IFN-α) and nucleoside analogues. PEGylated IFN-α (PEG-IFN-α) can lead to the disappearance of HBsAg in 10% or fewer of the patients treated, but it often comes with significant side effects.^20,21^ Nucleoside analogues suppress HBV replication but are unable to inhibit cccDNA in infected hepatocytes. Hence, this DNA triggers unrestricted viral protein production and viral replication, damages the immune system, and leads to chronic liver damage due to HBV.^22^ These limitations suggest the need for new therapies with the potential to provide long-term viral clearance. Therapeutic vaccines have, however, been ineffective in clinical trials due to inefficient uptake of antigens as well as weak immune response. Successful immunotherapy strategies must address the restoration of normal T-cell immunity, boosting the levels of anti-HBs antibodies, and subsequent elimination of the virus antigens and DNA in patients with chronic hepatitis B.^22-29^

 HBsAg-based nanovaccines have been introduced recently as a new promising approach to break through these challenges.^22-30^ Traditional vaccine preparations typically rely on alum adjuvants; however, adjuvant-free immune responses can be enhanced using nanovaccine delivery systems.^31,32^ Polymeric nanoparticle-aided controlled degradation provides the capability to present antigens for a longer duration, which leads to increased immune activation.^22-29,33^ Nanoparticles help cells take in antigens mainly through a process called endocytosis, especially pinocytosis, because they are similar in size to parts of the cells.^34-37^ These vaccine platforms have unique benefits, like mimicking real infections, improving how APCs absorb antigens, and co-delivering antigens and adjuvants to the same APCs, which boosts the immune system’s response.^22-29^ Because nanovaccines are very small, they can easily migrate to lymph nodes, which are the key areas of immune response and thus play a crucial role in the vaccine working even better.^22,24,26,38^

 With such advances, HBsAg-based nanovaccines may be a therapeutic treasure trove for the eradication of CHB. Despite this, to our knowledge, no published review is available that collates the merits and demerits of HBsAg-based therapeutic nanovaccines to clear HBV. Therefore, the current systematic review aimed to comprehensively evaluate the therapeutic effectiveness of this platform in increasing antibody yields, HBsAg/DNA clearance, induction of T-cell responses, and enhancing hepatitis B core antigen (HBcAg)-positive hepatocytes. With the application of nanotechnology, novel vaccine strategies may deliver an effective cure for chronic HBV infection, one of the most important unmet medical needs.

## Methods

###  Study Selection and Eligibility Criteria 

 We prepared this systematic review according to the PICO (Population, Intervention, Comparator, Outcome) strategy to assess the efficacy and safety of HBsAg-based nanovaccines in HBV treatment. The population consisted of human and animal models immunized with nanovaccine products with HBsAg as the primary immunogen. We included studies utilizing control groups, such as placebo, untreated controls (e.g., saline solution), free antigen, and adjuvanted controls. The primary objective was to ascertain the extent of HBsAg and HBV DNA load reduction achieved by the therapeutic vaccine. Secondary objectives were to trigger the body to generate anti-HBs, enhance the T-cell response, and decrease the level of HBcAg-positive hepatocytes. We also demonstrated how the nanovaccines influenced the liver tissue, alanine transaminase (ALT), and aspartate transaminase (AST) liver function tests. We considered experimental studies that had examined HBsAg-based nanovaccines, either constructed from the HBsAg protein or the HBsAg-encoding mRNA. The review involved randomized controlled trials (RCTs), cohort studies, case-control studies, and preclinical animal studies of HBsAg-based nanovaccines. We considered only English-language articles from 2015 to 2025. The study was approved by the Ethics Committee of Iran University of Medical Sciences (No. IR.IUMS.REC.1404.015).

###  Search Strategy

 A systematic review of the literature was performed according to the PRISMA guidelines.^39^ On February 18, 2025, we conducted detailed searches in the PubMed, Scopus, and Web of Science databases. The search was limited to English-language literature from 2015 to 2025. The initial search strategy had the following terms: (“Hepatitis B” OR “HBV”) AND (“Hepatitis B Surface Antigen” OR “HBsAg”) AND (“Nanovaccine” OR “Nanoparticles”) AND (“Treatment” OR “Therapy” OR “Therapeutic” OR “Cure”) AND (“Liver Cirrhosis” OR “Chronic Infection”). Boolean operators were then used to sequentially narrow down the search to maximize its sensitivity and specificity. Manual browsing of the found articles’ reference lists was also undertaken to reveal other relevant studies.

###  Data Extraction

 Two reviewers independently extracted data utilizing specifically created structured data extraction forms for this review. Discrepancies were resolved through consensus with a third evaluator. We contacted the relevant authors, including up to two follow-up emails if needed, to elucidate ambiguous or absent data. Information was extracted from tables, figures, the main text, and supplementary materials.

 In preclinical experiments, we collected detailed information on animal models of CHB, vaccine composition, nanoparticle characteristics (e.g., size, synthesis method, encapsulation efficiency (%EE), and release profile), administration route and dosage regimen, experimental group design, immunological parameters (e.g., anti-HBs antibody titer, clearance of viral antigens, levels of HBV DNA, and T-cell responses including IFN-γ production), and histological recovery of the liver.

 Extracted data from the clinical trials included patient profile, dosing regimen of the nanovaccine, randomization groups, and study design. Clinical endpoints included rates of seroconversion, viral clearance, and ALT and AST test normalization.

 The information gathered encapsulated the chemical and physical characteristics of nanovaccines, immunization timetables adopted, and effectiveness outcomes reported using real clinical setups and preclinical trials.

###  Risk of Bias Assessment

 Risk of bias assessments were used to interpret the robustness of study findings and guide the synthesis of evidence. This was independently assessed by two reviewers, with disagreements resolved through consultation with a third reviewer. The results of human and animal studies were presented separately to account for study-type differences. The following tools were applied: (i) SYRCLE’s risk of bias tool for animal studies; (ii) ROBINS-I tool for non-randomized clinical studies, and (iii) cochrane risk of bias 2 (RoB 2) tool for randomized controlled trials. Bias assessment data are depicted in Figure 1.

**Figure 1:**
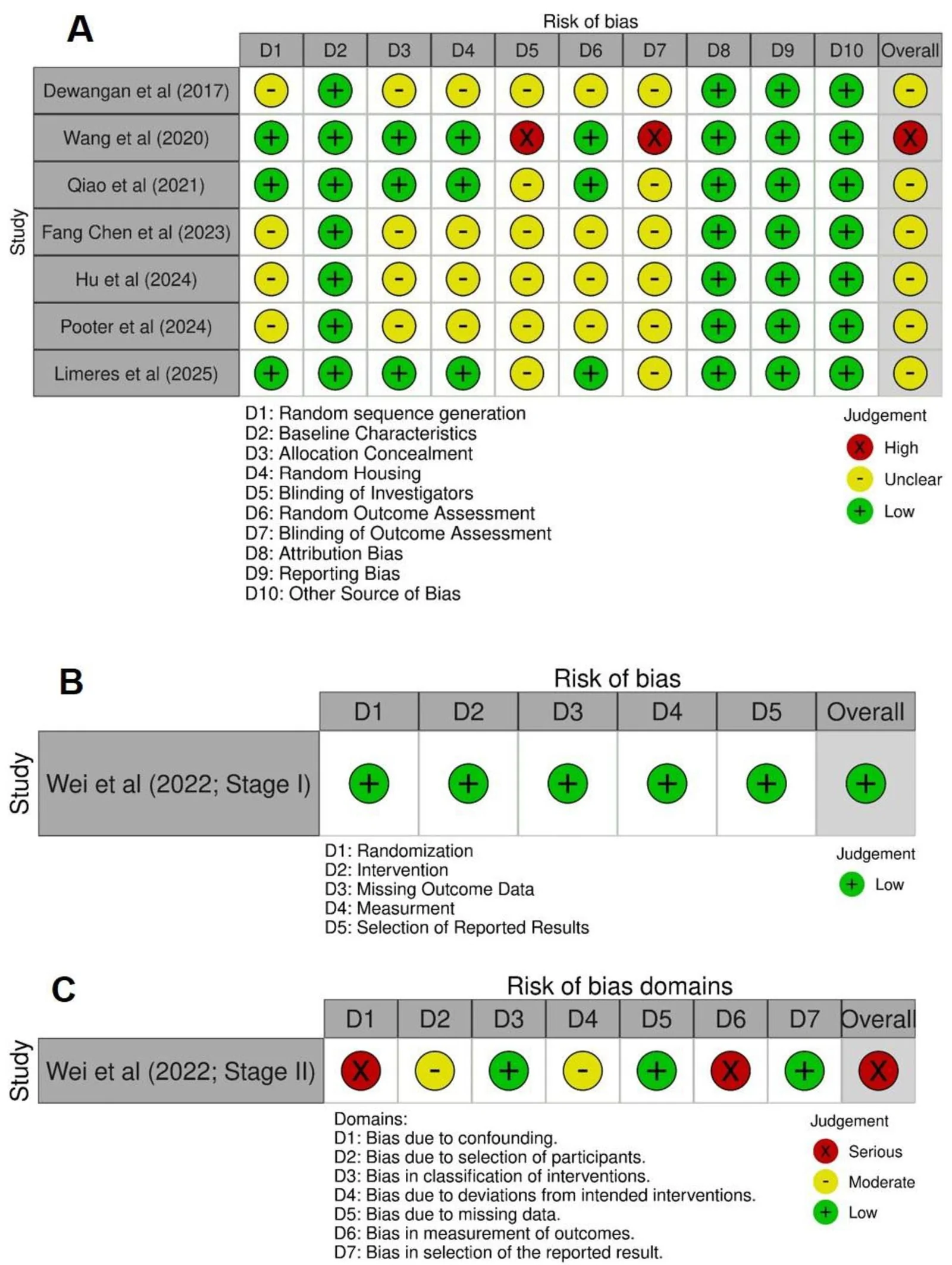
Risk of Bias Assessment for Included Studies. (A) SYRCLE's Risk of Bias Tool for animal studies. The studies were assessed in ten domains (D1–D10), and their assessment is depicted as low risk (green), unclear risk (yellow), or high risk (red). (B) the Cochrane Risk of Bias 2 (RoB 2) tool for randomized trials, in which bias was evaluated in five domains (D1–D5) using the same color coding. (C) ROBINS-I assessment for non-randomized observational clinical studies, in which bias was evaluated in seven domains (D1–D7) using the same color coding

## Results

###  Results on Study Selection 

 The study selection procedure is depicted in the PRISMA flow diagram (Figure 2). A total of 361 articles were identified through electronic database searches: PubMed/Medline (n = 48), Scopus (n = 133), and Web of Science (n = 180). We screened 289 articles based on title and abstract, following the predefined inclusion and exclusion criteria, after removing duplicate records (n = 72). Of these, 11 studies met our inclusion criteria and were included in the final analysis. After full-text analysis, three studies were excluded since they were on HBeAg (n = 2) and had no effect on viral clearance (n = 1). Finally, we found eight studies comparing the therapeutic outcomes of HBsAg-based nanovaccines that met all our inclusion criteria. Data from preclinical studies are summarized in Tables 1 and 2, while the results obtained from the only clinical study are presented in Table 3.

**Figure 2:**
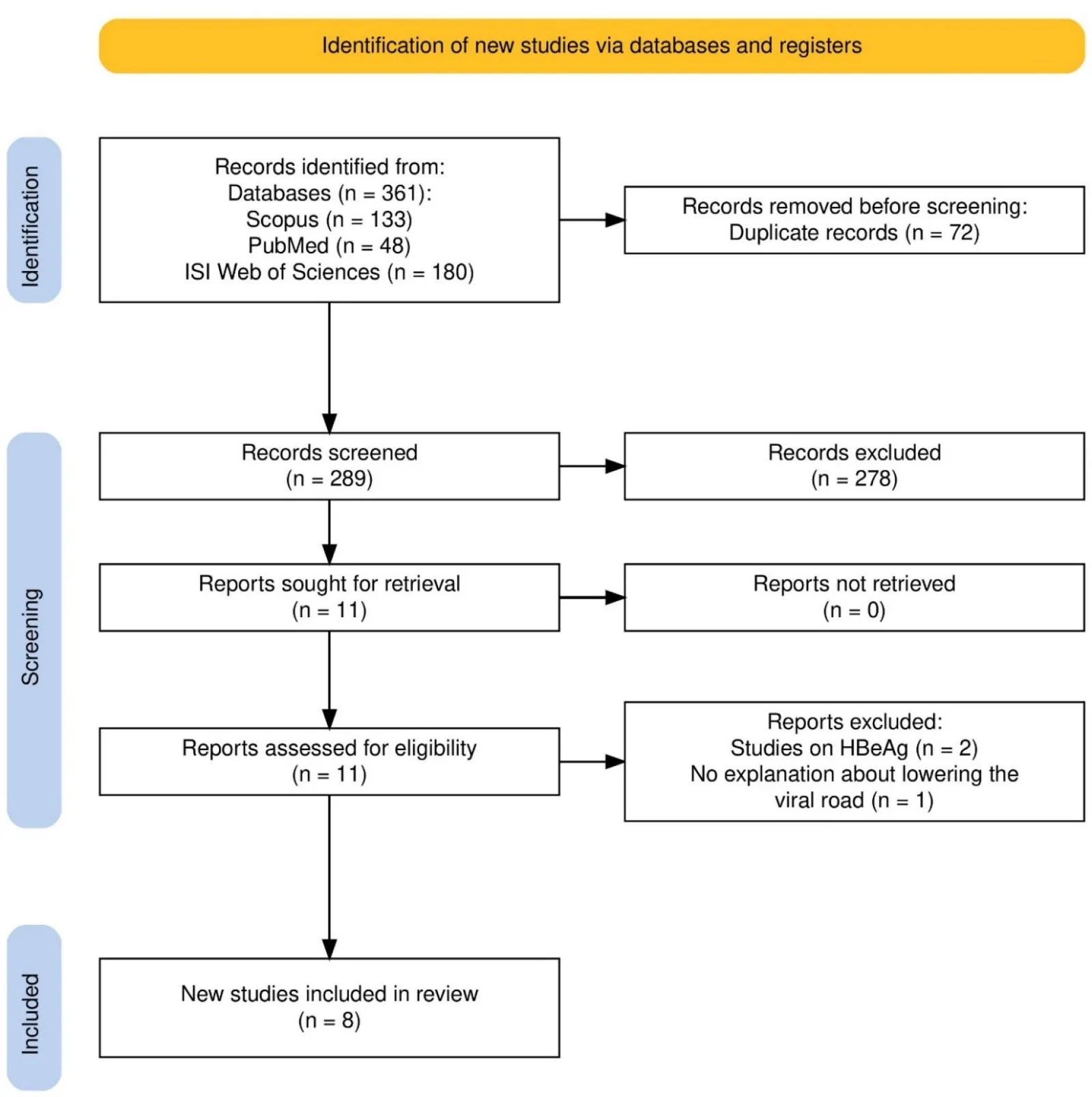
PRISMA flow chart summarizes the study selection procedure

**Table 1:** Characteristics of HBsAg-based nanovaccine platforms in preclinical HBV models

**Study**	**Model**	**Nanovaccine**	**Synthesis method**	**Size / Charge**	**EE (%)**	**Release profile / Stability**	**Route**
Dewangan et al.^23^	Chimera mice	PLGA NP–HBsAg + TT	W/O/W emulsion solvent evaporation	262–274 nm	High	~90–92% in 36 days	I.M.
Wang et al.^24^	Naïve wild-type C57BL/6 mice and HBV carrier mice	Ferritin NP–preS1 vaccine	SpyTag/SpyCatcher conjugation	NR	NR	Long-lasting	S.C.
Fang Chen et al.^25^	HBV-carrier mice (rAAV-HBV1.3)	DOTAP-assisted CLAN CpG nanoparticles + rHBsAg	A microfluidic technology using the Advanced-Flow Reactors (with PEG5K-b-PLGA10K and DOTAP)	71–104 nm / + ve	71–95%	NR	I.P., S.C.
Qiao et al.^22^	AAV/HBV model	Chitosan–Heparin NPs (NSG or NCG)	Electrostatic	~65–67 nm	High	Stable 35 days	S.C. (footpad)
Hu et al.^26^	C57BL/6J + pAAV/HBV1.2	PP-SG (HBsAg + c-di-GMP)	Self-assembly method using solvent evaporation and nanoprecipitation	30–40 nm / ~0 mV	HBsAg (~84.3%); c-di-GMP (~78.6%)	Sustained (72 h)	S.C.
Limeres et al.^29^	C57BL/6 mice rAAV8-1.3HBV model	mRNA encoding HBsAg in LNP	Microfluidic mixing (NanoAssemblr Ignite); Lipid mix: Genvoy-ILM^TM^ (50% ionizable lipid, 10% DSPC, 37.5% cholesterol, 2.5% PEG-lipid)	80–90 nm	~95%	NR	I.M.
Pooter et al.^28^	AAV-HBV mouse model	mRNA encoding HBcAg, Pol, and preS2-S in LNP	Microfluidic mixing	80–100 nm	> 90%	Controlled release post-endocytosis; No significant burst release before cellular uptake (typical characteristic of LNPs)	I.M.

EE, Entrapment Efficiency; I.M., intramuscular; S.C., subcutaneous; I.P., intraperitoneal; NR, Not Reported; pAA-pEPEMA, (poly(2-aminoethyl methacrylate hydrochloride)-b-poly(ethylene glycol)-b-poly(2-(dimethylamino)ethyl methacrylate));

**Table 2:** Immunogenicity and therapeutic outcomes of HBsAg-based nanovaccines in HBV clearance

**Nanovaccine**	**Antibody response**	**Viral clearance**	**T-cell activation**	**Liver histology**	**Mechanism of control**	**Ref**
PLGA–HBsAg + TT	↑ anti-HBs (35,621 mIU)	↓ HBsAg, HBcAg, HBV DNA (day 18)	↑ Th1/IFN-γ and CTLs; restored PBMCs	↓ HBcAg + hepatocytes	Cytolytic & non-cytolytic (IFN-γ, CTLs, NKs)	Dewangan et al.^23^
Ferritin–preS1	600 × ↑ anti-preS1; high avidity IgG1 isotypes, ↑ anti-HBs (long-lasting)	↓ HBsAg, HBV DNA, cccDNA	↑ IFN-γ-producing preS1-specific T cells; ↑ Th1-biased response	↓ HBcAg + hepatocytes; ↓ cccDNA No damage; ALT/AST normal	DC/macrophage targeting, ADCC, B cell activation	Wang et al.^24^
CLAN–CpG + rHBsAg	↑ IgG2a (Th1-biased response)	↓ HBsAg, HBeAg, HBV DNA (day 28)	↑ DCs (CD40/CD80/CD86), ↑ IFN-α; ↑ NK/T cell implied	↓ Inflammation, ↓ ↓ HBcAg + hepatocytes	TLR-9 → IFN-α, DC maturation	Chen et al.^25^
NSG + NCG	↑ anti-HBs; 80% anti-HBs seroconversion; ↑ IgG2a/IgG1 ratio (Th1-biased response)	90% HBsAg clearance; ↓ HBV DNA	↑ IFN-γ, IL-2, IL-4; strong CTL response; ↑ DCs (CD40/CD80/CD86; IL-12)	↓ HBcAg + hepatocytes; Normal AST/ALT; A Restoration of T cell response in hepatocytes	APC uptake↑, LN targeting, Th1/CTL bias	Qiao et al.^22^
PP-SG (c-di-GMP)	Durable anti-HBs IgG; memory on rechallenge	↓ HBsAg, DNA, RNA, HBcAg; persistent on rechallenge	↑ CD4 + , CD8 + ; ↑ cDC1 and cDC2	No hepatological damage	cGAS–STING activation, IRF3, memory recall	Hu et al.^26^
mRNA encoding HBsAg in LNP	- Strong anti-HBsAb	Rapid HBsAg and HBV DNA clearance	↑ CD8⁺ and CD4⁺ T cells (↑ IFN-γ, TNF-α, IL-2)	NR	Robust humoral and cellular immune activation	Limeres et al.^29^
mRNA encoding HBcAg, Pol, and preS2-S in LNP	- Anti-HBsAb (↑ after 2nd/3rd dose) - Anti-HBeAb (↑ after 3rd dose)	- HBsAg ↓ (by 1.0–1.7 log10 IU/mL in 50% of vaccinated mice); - HBeAg ↓ transiently (0.4–0.7 log10 IU/mL); - No significant DNA reduction	CD8⁺ T cell ↑ (Pol1/Pol2 epitopes); Moderate CD4⁺ T cell response	- No change in HBcAg⁺ hepatocytes - No ALT elevation	- Partial viral suppression by CD8⁺ T cells; - Antibody-mediated antigen reduction (incomplete clearance)	Pooter et al.^28^

↑ = increased; ↓ = decreased; TT, Tetanus toxoid; Th, T helper cells; CTL, cytotoxic T lymphocyte; DC, dendritic cell; NK, natural killer; LN, lymph node; NR, Not Reported; TLR, Toll-like receptors; APC, Antigen-presenting cell; cGAS-STING, cyclic GMP-AMP synthase–stimulator of interferon genes; ALT, Alanine transaminase; AST, Aspartate transaminase.

**Table 3:** Clinical trials on therapeutic HBsAg-based nanovaccines

**Author (Year, Trial ID)**	**Study design**	**Population (n)**	**Mean age** **(year)**	**F:M ratio**	**Dosing schedule**	**Treatment arms**	**HBeAg seroconversion (Week 76)**	**Combined endpoint†**	**Virologic response‡**	**Biochemical test§**	**HBeAg seroconversion (Extended)**	**HBV DNA<2000 IU/mL (Extended)**
Wei et al.^27^ NCT00869778	*Stage 1:* 76-week RCT; *Stage 2:* 68-week open-label extension	*Stage 1:* 360 (HLA-A2 + , HBeAg + CHB); *Stage 2:* 209 (183 continued, 26 follow-up)	27	26.4%: 73.6%	At weeks 0, 4, 8, 12, 20, 28 (S.C.)	600 µg εPA-44	33.6% (40/119)	14.3% (17/119)	19.3% (23/119)	NR	NR	NR
						900 µg εPA-44	38.8% (45/116)	18.1% (21/116)	19.8% (23/116)	48.3% (56/116)	22.1% (30/136)	23.5% (32/136)
						Placebo	20.2% (24/119)	5.0% (6/119)	13.4% (16/119)	34.5% (41/119)	0%	5.9% (8/136 at BL)

RTC, Randomized controlled trial; HLA, Human leukocyte antigen; NR, Not reported; S.C., Subcutaneous; CHB, Chronic Hepatitis B; BL, Baseline. ^†^Combined endpoint: HBeAg seroconversion + ALT normalization + HBV DNA < 2000 IU/mL. ^‡^Virologic Response = HBV DNA < 2.93 × 10⁴ IU/mL. ^§^BR: ALT normalization.

###  CHB Animal Models

 Wild-type mice (e.g., BALB/c and C57BL/6J) are generally used to evaluate the immune responses against HBV recombinant antigen-based vaccine candidates, e.g., rHBsAg and rHBcAg. Researchers monitored the immunogenicity of these vaccines regarding their antibody seroconversion rates, T-cell responses (e.g., CTL responses), and cytokine production.^22-29^

 To simulate CHB, transgenic mice expressing the HBV genome have been developed. While useful, early expression of HBV antigens induces central immune tolerance, limiting their suitability for studying therapeutic interventions and viral clearance.^40-43^ Alternatively, novel animal models have been established by hydrodynamic injection (HDI), where the HBV genome is directly delivered into the mouse bloodstream.^44^ One such technique is the application of adeno-associated virus (AAV) vectors to stably express HBV for inducing chronic infection.^45^ As AAV8 is highly hepatotropic, the rAAV8-1.3HBV system has been widely used to study the antiviral efficacy of diverse drug product candidates against HBV. The HDI of the HBV genome with AAV vectors was found to be able to efficiently induce long-term virus persistence; therefore, this is a good tool to explore how the body can tolerate HBV. HDI models are not faultless, though, as they are unable to mimic the extremely high levels of HBV viremia observed in patients with CHB.^40^

 Moreover, infant or newborn mice utilized in studies to evaluate CHB did not establish chronic infections since the immune system matured too early and blocked long-lasting virus persistence. However, mice that had been injected with recombinant AAV vectors delivering the HBV genome (AAV/HBV) were discovered to be producing sustained virus carriage for more than 30 weeks, making them candidate models for explaining how HBV infection is maintained. Then, the recombinant AAV-HBV models, particularly in C57BL/6 mice, keep a steady level of HBV in the blood and can be used to examine how the immune system reacts to long-term infection and to explore new treatment options.^40^

 Finally, researchers can also study the treatments of HBV in chimera mice, or genetically engineered mice that mimic human immune reactions. Trimera or humanized mice is also another name for the model. The model allows researchers to study the reaction of the human immune system against HBV antigens, like HBsAg, by transplanting human blood cells into infection-fighting, compromised mice. The strategy is designed to stimulate human T cells and B cells after HBV vaccination, making it possible to observe the vaccine efficacy and clearance of HBV. Immunization of chimera mice with HBsAg causes the generation of immune complexes that make antigen-presenting cells present more efficiently, thus causing activation of more T cells. The system is most effective for the measurement of antibody responses and the identification of antiviral drugs.^23^

###  Preclinical Studies: Design Chemistry and Therapeutic Potency

####  Protein-Based Nanovaccines

#####  PLGA (Poly(lactic-co-glycolic Acid)) Nanoparticles

 An HBsAg-based nanovaccine was prepared using a solvent evaporation method in a water-oil-water (W/O/W) emulsion, which produced particles in the size range of 262 ± 1.4 to 274 ± 9 nm. The particle size and encapsulation efficiency were affected by different concentrations of PLGA and polyvinyl alcohol (PVA), the ratio of aqueous to organic phase, and the homogenization rate. Increasing the homogenization rate and PVA concentration resulted in the production of smaller, more stable nanoparticles with higher encapsulation efficiency. Antigen release was sustained and gradual, with 90–92% of the antigen released within 36 days; part of the initial release was attributed to antigen bound to the particle surface.

 To evaluate the immunological and antiviral efficacy of the nanovaccine, a chimeric xenograft model was used. Three treatment groups were defined as group I (BMT + PBMC + HBsAg nanoparticles), group II (BMT + PBMC + tetanus toxoid + saline), and group III (BMT + PBMC + tetanus toxoid + HBsAg nanoparticles). The initial viral load was similar in all groups (mean 3.8 × 10^6^ copies/ml). At day 18, groups I and III showed a decrease in viral load (0.35 × 10^6^ and 0.25 × 10^6^ copies/ml). Anti-HBs antibody titer was correlated with viral clearance, so that the highest titer was observed in the third group (35621 ± 4.6 mIU/mL).

 PCR results showed a gradual decrease in HBV-DNA in serum and liver tissues from the vaccinated groups. Histological and immunohistochemical analyses also confirmed a significant decrease in HBcAg-positive liver cells after vaccination. In addition, stronger interferon-gamma-producing T cell responses, more effective CTL activity, and increased NK cell responses were observed, all indicating restoration of suppressed Th1 immunity and effective virus suppression.^23^

####  Cationic Lipid-Assisted Nanoparticles (CLANs)

 Six types of CLAN nanoparticles were synthesized using the microfluidic emulsification method and PEG5K-b-PLGA10K polymer, which included three types of cholesterol-based lipids (CL3, CL7, BHEM-Chol) and three types of alkyl chain lipids (AL6, AL7, DOTAP). Among them, DOTAP-CLAN nanoparticles (loaded with CpG) showed the best properties, with a high CpG entrapment efficiency (up to 95.3%), sizes of 71.0–103.9 nm, a positive zeta potential, and stability in serum for more than 72 h.

 Therapeutically, a prime-boost regimen of the vaccination was employed in HBV-carrier mice to evaluate the effectiveness of a DOTAP-mediated CLAN delivering CpG ODN with rHBsAg for viral clearance. In the prime phase, DOTAP-CLANCpG was administered intraperitoneally at 1 mg/kg (100 µL/mouse) daily for three consecutive days. This was followed by four once-daily subcutaneous injections of the same preparation with rHBsAg at 0.1 mg/kg. Two weeks later, the entire vaccination regimen was again administered as a booster immunization. Viral and immunological characteristics were quantified by collecting blood weekly. Mice treated with the DOTAP-assisted CLAN CpG and rHBsAg combination exhibited a marked reduction in serum HBsAg and HBeAg levels compared to control groups, indicating potent suppression of viral antigenemia. On day 28, quantitative PCR observed the removal of vaccine-induced HBV DNA in serum from the vaccinated group, while other groups (free CpG ODNs, unloaded CLANs, and PBS controls) were incapable of doing so.

 Histopathological studies showed the absence of HBcAg expression in liver cells. Moreover, the vaccinated group showed a reduction in inflammation and liver tissue damage. Also, blood biochemical indices (ALT, AST, total bilirubin) were significantly reduced, indicating improved liver function and reduced hepatocellular damage. In addition, DOTAP-CLANCpG had high uptake by plasmacytoid dendritic cells (pDC) and common dendritic cells (cDC), which was evidenced by a significant increase in the expression of maturation markers (CD40, CD80, CD86) in splenic dendritic cells within 12 hours after injection. Intravenous injection of this nanovaccine also resulted in increased serum interferon-alpha levels and strong activation of the Toll-like receptor-9 (TLR-9)-dependent innate immune pathway.^25^

####  Nanovaccines Loaded with rHBsAg, rHBcAg, and CpG

 HBcAg has been reported to have the capability to stimulate B cells to perform optimally as initial APCs and to have a synergetic effect in stimulating antibody and cellular immunity when used in combination with HBsAg.^16,46^ This antigen was thus incorporated into nanovaccine preparation by Qiao et al.^22^ The nanovaccines were prepared using a multi-inlet microfluidic mixer, which mixed chitosan, heparin, CpG, and either rHBsAg or rHBcAg. The electrostatic interaction between positively charged chitosan and negatively charged heparin at pH 4.5 enabled the antigen and adjuvant to be encapsulated into nanoparticles. Therefore, two preparations, i.e., NSG (rHBsAg + CpG) and NCG (rHBcAg + CpG), were formulated. The physicochemical parameters of both the nanoparticles proved to be favorable, with diameters of 67.1 nm (NSG) and 64.9 nm (NCG) under the ideal rate of flow of 10 mL/min, ensuring uniformity as well as less polydispersity. The nanovaccines had adequate encapsulation efficiency, a 24-hour prolonged release of antigens and adjuvants, and adequate stability for 35 days. Compared with the free antigen-adjuvant mixtures, the compositions significantly promoted macrophage uptake of the formulations and yielded the controlled release benefit required for immune activation induction.

 In an AAV/HBV mouse model, three subcutaneous doses of the NSG + NCG nanovaccine were administered on days 0, 14, and 28. This regimen resulted in a significant reduction in serum levels of hepatitis B virus surface antigen (HBsAg). In addition, 80–90% of mice achieved positive anti-HBs seroreactivity, and HBsAg clearance was observed. Moreover, an increase in the frequency of anti-HBs antibody-secreting B cells and IFN-γ was reported.

 Histological examination of post-treatment liver samples showed a lack of HBsAg- and HBcAg-positive hepatocytes in the vaccine group, while the control group showed a strong presence of these antigens. Serum AST and ALT levels were within normal limits. Mechanism-wise, these nanovaccines enabled an efficient uptake by APCs. The addition of CpG as a TLR-9 receptor agonist resulted in a strong stimulation of innate immunity in favor of Th1 responses, while the presence of HBcAg alongside HBsAg synergistically stimulated B cells and enhanced T cell responses. Sustained antigen release and retention in lymph nodes provided long-term immune stimulation, which collectively led to the breakdown of immune tolerance, clearance of viral antigens, and activation of HBV-specific cytotoxic T lymphocytes, ultimately leading to inhibition of viral replication and immune reconstitution.^22^

####  Lymph Node-Targeted STING Agonist Nanovaccine (PP-SG)

 Hu et al.^26^ developed a novel nanovaccine platform, PP-SG, consisting of HBsAg coupled with the STING agonist, i.e., cyclic diguanylate monophosphate (c-di-GMP). This combination was made using a synthetic amphiphilic diblock copolymer, poly(2-aminoethyl methacrylate hydrochloride)-b-poly(ethylene glycol)-b-poly(2-dimethylaminoethyl methacrylate) (pAA-pEPEMA). The nanoparticles were synthesized via a solvent evaporation and nanoprecipitation method, yielding homogeneous spherical particles with a size of 30–40 nm and a nearly neutral zeta potential, which is ideal for lymph node targeting and antigen uptake.

 In a mouse model of chronic HBV infection (pAAV/HBV 1.2), subcutaneous administration of PP-SG (containing 1 μg of HBsAg weekly for three times) resulted in significant reductions in serum HBsAg and HBV DNA levels. Significant reductions in HBV DNA and intrahepatic RNA transcripts (including 3.5 kb RNA) as well as in HBcAg expression were also observed. These effects were accompanied by strong anti-HBs IgG seroreactivity and the development of stable immune memory after re-exposure to the virus. At the same time, no hepatotoxicity or systemic side effects were observed, indicating the appropriate safety of this nanovaccine. From the perspective of mechanism of action, PP-SG nanovaccine effectively enhanced antigen presentation and immune responses of helper (CD4 ^+^) and cytotoxic (CD8 ^+^) T cells, especially through the stimulation of cDC1 and cDC2 subpopulations, by reactivating the suppressed cGAS-STING pathway and phosphorylated interferon regulatory factor type 3 (p-IRF3) signaling in dendritic cells.^26^

####  PreS1 Domain as a Target for Nanovaccine Design

 Evidence from several studies affirms the superior therapeutic promise of the preS1 domain of HBV large surface antigen compared with conventional HBsAg-based interventions. The preS1 domain promotes HBV entry by interacting with the sodium taurocholate co-transporting polypeptide (NTCP) receptor and is a target for neutralizing monoclonal antibodies.^47,48^ These antibodies effectively induce immune-mediated removal of infected hepatocytes by antibody-dependent cell-mediated cytotoxicity (ADCC) and phagocytosis.^49^ Clinically, the appearance of anti-preS1 antibodies correlates with HBV early clearance, indicating their therapeutic and prognostic value.^50^ Notably, immune tolerance to preS1 is much poorer in CHB patients than that to HBsAg; thus, it may be a preferable target to stimulate vigorous immune responses by nano-vaccination.^17,51^ Yet, preS1 is of very low immunogenicity during natural infection and conventional vaccination, where new platforms have been made to release its maximal immunostimulatory potential.^17,24,51^ Using the ferritin-based nanoparticle platform engineered via the SpyTag/SpyCatcher system, the preS1 domain of HBV large surface antigen was covalently displayed to enhance immunogenicity. This formulation (ferritin NP–preS1) induced a 50–150-fold increase in anti-preS1 antibody titers compared to soluble preS1, with sustained antibody presence up to day 235 and booster-enhanced titers reaching 600-fold elevation.^24^

 Therapeutic vaccination with this nanovaccine in AAV-HBV1.3-infected mice significantly reduced serum levels of preS1 antigen, HBsAg, and HBV viral DNA. In addition, in 57% of treated animals, viral DNA and HBsAg levels reached undetectable levels, and anti-HBs antibody titers increased. According to liver histological analysis, a significant reduction in HBcAg expression and cccDNA levels was reported. Interestingly, this platform induced no hepatotoxic impacts, supported by normal levels of ALT and AST. Mechanistically, the vaccine induced a strong Th1-biased cellular immune response characterized by increased IFN-γ production from preS1-specific T cells in the liver, spleen, and lymph nodes. Inhibition of IFN-γ abolished the virus clearance effect. These nanoparticles were targeted to APCs expressing the SIGNR1 receptor, specifically resident DCs and lymph node macrophages. Dendritic cells play a key role in the induction of T follicular helper (Tfh) cells, which elicit antibody responses in the germinal center. Also, CpG-induced expression of the CXCR5 receptor in macrophages allowed follicular migration and B-cell activation—a novel CXCR5-dependent pathway that is critical for efficient antigen presentation.^24^

####  mRNA-Based Nanovaccines

#####  HBsAg-Encoding mRNA in Lipid Nanoparticles (LNPs) 

 Limeres et al.^29^ formulated HBsAg-encoding mRNA vaccines in two forms, wild-type and MHC-I signal peptide-tagged (SP-HBsAg), in LNPs using the NanoAssemblr Ignite^TM^ and GenVoy-ILM^TM^ systems. These nanoparticles had a size of 80–90 nm, a particle size distribution index of less than 0.05, and an encapsulation efficiency of approximately 95%. Intramuscular injection of 5 μg of mRNA-LNP into C57BL/6 mice resulted in early activation of CD69 ^+^ T cells and CD80 ^+^ /CD86 ^+^ dendritic cells. Compared with ENGERIX^®^-B, both mRNA-LNP formulations induced higher titers of anti-HBsAg (IgG), with SP-HBsAg having a dominant Th1-type response with an IgG2c/IgG1 ratio of greater than 1. The vaccine also increased CD4 ^+^ and CD8 ^+^ specific T cell responses (IFN-γ ^+^, TNF-α ^+^, IL-2 ^+^). After challenge with rAAV8-1.3HBV, vaccinated mice showed complete clearance of HBsAg and maintenance of anti-HBsAg IgG levels, whereas controls had high antigen levels. The mRNA-LNP with SP-HBsAg also reduced plasma HBeAg levels below the limit of detection. Liver histological evaluation was not performed in this study. The mechanism of action involved overcoming immune tolerance by potently activating a Th1-type cell response and enhancing antigen presentation via MHC-I targeting.^29^

####  LNPs Containing mRNAs Encoding HBcAg, Polymerase (Pol), and preS2

 Pooter et al.^28^ examined the antiviral activity of an LNP-based mRNA vaccine encoding HBcAg, pol, and preS2-S antigens in healthy mice and mice infected with AAV-HBV. The mRNA strands were encapsulated individually, in combination, or co-encapsulated in a lipid particle to maximize delivery and immune activation. These vaccines induced HBV-specific multifunctional CD8 ^+^ and CD4 ^+^ T cell responses. Moreover, both HBsAg- and HBeAg-specific antibodies appeared after vaccination, particularly after the third dose. Serum HBsAg levels decreased by 1.0 to 1.7 log_10_ IU/mL in 50% of vaccinated mice. A transient decrease in HBeAg levels (0.4 to 0.7 log_10_ IU/mL) was also observed in this same group, but a sustained decrease in HBeAg was not achieved. While serum HBsAg levels were decreased, immunohistochemical examination of the liver showed only a slight decrease in HBsAg-positive hepatocytes. Furthermore, there was no significant difference in the number of HBcAg-positive cells between vaccinated groups and saline-treated controls. ALT levels did not change, indicating minimal clearance or damage to hepatocytes. The presence of the envelope antigen (preS2-S) in the mRNA formulation enhanced the immunogenicity and antiviral activity of the vaccine.^28^

###  Clinical Effectiveness of Therapeutic HBsAg-Based Nanovaccines

 A two-stage phase II clinical trial (NCT00869778) was conducted to determine the effectiveness and safety of εPA-44, a liposome nanoparticle vaccine, in patients with HBeAg-positive chronic hepatitis B. The study included a 76-week randomized, double-blind, placebo-controlled stage 1 and a 68-week open-label extension stage 2 in 15 Chinese centers (Data is summarized in Table 3). At week 76, results indicated that HBeAg seroconversion occurred significantly more often in the εPA-44 groups compared with placebo. HBV DNA suppression was higher in the 900 µg (19.8%) and 600 µg (19.3%) groups compared to placebo (13.4%), but not statistically significant at week 76. ALT normalization rate was significantly greater with 900 µg εPA-44 (48.3% (56/116)) compared to placebo (34.5% (41/119)). The combined end-point (HBeAg seroconversion + normalization of ALT + HBV DNA < 2000 IU/mL) was considerably higher in both 600 µg and 900 µg εPA-44 groups compared to placebo. Multi-factor logistic regression analysis found age, initial HBeAg level, and εPA-44 dose to be predictors for seroconversion.^27^

 For stage 2, 209 patients continued into the open-label extension. In non-responders to HBeAg seroconversion by week 76, further 900 µg εPA-44 treatment enhanced outcomes: (i) HBeAg seroconversion rate was increased from 0% to 22.1% (30/136; *P* < 0.001) and (ii) Percentage of patients with HBV DNA < 2000 IU/mL increased from 5.9% to 23.5% (*P* < 0.001). No serologic relapses (0/20) were observed in those with serologic and virologic responses, and they were followed up without further treatment. The tolerability of εPA-44 was similar to placebo at both phases. Adverse events (AEs) were mostly mild or moderate, e.g., local reaction at the injection site and reversible increases in ALT. No serious treatment-emergent AEs occurred, and no patients withdrew due to severe AEs.^27^

## Discussion

 Chronic hepatitis B remains a severe global health problem, largely due to the persistent presence of cccDNA and profound depletion of HBV-specific immune competence. Current antiviral therapy, although capable of keeping viral replication in check, is not able to clear cccDNA reservoirs or fully restore functional immune control and therefore limits the realization of a sterilizing cure. Here, HBsAg-based nanovaccine generation is a groundbreaking approach towards a functional cure, with the characteristics of sustained HBsAg loss, with or without seroconversion of anti-HBs, and sustained HBV DNA suppression. Through this systematic review, we evaluated the therapeutic promise of various HBsAg-based nanovaccine platforms, including protein- and mRNA-based systems. Our analysis highlighted noteworthy distinctions among nanoparticle compositions, antigen delivery methods, immune activation profiles, and overall therapeutic outcomes.

 All reviewed nanovaccines showed high encapsulation efficiencies (> 70%), indicating efficient antigen loading strategies.^22-29^ Particle size, however, differed considerably among formulations and affected biodistribution and lymphatic targeting. PLGA nanoparticles (~270 nm) were comparatively larger than ferritin-based (70–100 nm) and PP-SG (~30–40 nm) nanoparticles.^23,25,26^ Smaller particle sizes, especially those < 100 nm, are beneficial for passive drainage into lymph nodes and increased uptake by APCs.^52,53^ Interestingly, LNP-formulated mRNA vaccines (80–90 nm) exhibited advantageous size distributions (low PDI < 0.05), which suggests better lymphoid trafficking and immunogenicity.^28,29^ Most of the vaccines took subcutaneous or intramuscular routes to administer the vaccine to induce maximal immune responses,^22-29^ but intraperitoneal administration was strategically used in a few experiments to further enhance immunogenicity.^25^

 All the nanovaccines reviewed here provoked a strong humoral response against the HBV antigens. In protein platforms, PLGA particles that encapsulated HBsAg and boosted with TT developed very high anti-HBs titers (~35,621 mIU on Day 18), indicating their function as an adjuvant delivery system.^23^ Likewise, ferritin-preS1 nanoparticles induced 600-fold IgG1 antibody of high titers.^24^ However, both DOTAP-guided CLAN CpG nanoparticles + rHBsAg and NSG + NCG co-administration induced very high IgG2a responses characteristic of Th1-type immunity.^22,25^ Interestingly, PP-SG nanovaccines demonstrated the eradication of serum HBsAg and intrahepatic HBV markers, signifying successful long-term viral clearance.^26^ Among mRNA vaccines, the HBsAg mRNA-LNP vaccine^29^ (Limeres et al., 2025) was superior to its pol-, HBcAg-, and preS2-S antigens-expressing competitors^28^ (Pooter et al., 2024) in inducing earlier, higher-titer anti-HBs titers and improved HBsAg seroclearance.^28,29^ Interestingly, the HBsAg mRNA-LNP vaccine was also better than the protein ENGERIX^®^-B vaccine, especially when looking at the IgG2c/IgG1 ratios, due to its immune response that favors Th1.^29^

 The key markers for therapeutic vaccination efficacy in HBV are HBsAg and HBV DNA reduction. Ferritin-preS1 immunization, application of PLGA particles, DOTAP-modified CLAN CpG nanoparticles, and PP-SG nanoparticles decreased serum HBsAg to a significant level.^23,25,26^ Co-administration of NCG and NSG nanvaccines could reduce viral clearance to 90%,^22^ while intrahepatic HBV markers and serum HBsAg were significantly disappeared by PP-SG nanovaccine therapy.^26^ In mRNA-based approaches, the HBsAg mRNA vaccine^29^ induced more pronounced reductions in serum HBsAg and HBV DNA than the tri-antigen mRNA vaccine^28^, further noting the strategic advantage of targeting PreS1 regions implicated in viral entry.^28,29^

 Successful therapeutic vaccination against CHB must restore HBV-specific T-cell responses. The majority of nanovaccines, independent of platforms, triggered strong Th1-type responses with robust IFN-γ secretion, CTL activation, and favorable CD4 ^+^ /CD8 ^+^ T-cell profiles. Protein-based nanoveaccines such as Ferritin-preS1 NPs and DOTAP-facilitated CLAN CpG nanoparticles robustly activated IFN-γ-secreting T cells.^24,25^ NSG + NCG nanovaccine remarkably induced multifunctional CD4 ^+^ and CD8 ^+^ T-cell responses with IL-2, IFN-γ, and IL-4 secretion, a hallmark of Th1/Th2 balanced response.^22^ Furthermore, PP-SG induced the activation of conventional dendritic cells (cDC1 and cDC2), which are important for cross-priming cytotoxic T lymphocytes.^26^ The mRNA vaccines also stimulated strong antigen-specific T-cell immunity. More polyfunctional and balanced T-cell activation (IFN-γ, TNF-α, IL-2) was achieved with the HBsAg mRNA vaccine compared to the tri-antigen mRNA vaccine, which was consistent with its improved viral clearance findings.^28,29^

 One of the significant concerns with therapeutic vaccines is the prevention of their hepatocellular injury. Fortunately, in the majority of studies, there was no increase in liver enzymes (ALT, AST) and no significant histopathological injury following immunization. Notably, ferritin-preS1 NP vaccine reduced intrahepatic HBcAg and cccDNA considerably without hepatotoxicity.^24^ DOTAP-coated CLAN CpG nanoparticles and NSG + NCG nanovaccine relieved hepatitis and hepatocyte viral antigen expression without liver toxicity.^22,25^ The PP-SG nanovaccine, while less potent in reducing HBcAg-positive hepatocytes, also showed a safety profile without liver toxicity.^26^ Although mRNA vaccine encoding HBcAg, pol, and preS2-S antigens could lower intrahepatic HBcAg without inducing any hepatic damage, liver histology data for another mRNA vaccine were unavailable,^28,29^ which is highly recommended to be evaluated by future studies.

 This review highlights the therapeutic potential of targeting the PreS1 domain to facilitate HBV entry into hepatocytes. The structural presentation of PreS1 via nanoplatforms, such as ferritin-based nanoparticles, markedly augmented long-term, high-avidity antibody responses and facilitated accelerated viral clearance.^24^ These findings underscore the significance of antigen design and confirm that conformationally accurate, multivalent PreS1-presenting vaccines possess the capability to surmount immune tolerance obstacles associated with CHB.

 Finally, the HBsAg-based nanovaccines can be translated into clinical settings. For example, in a two-stage phase II clinical trial, a liposomal nanoparticle vaccine demonstrated spectacular clinical benefits among HBeAg-positive patients with CHB. This nanoplatform, particularly at the dose of 900 µg, considerably improved rates of HBeAg seroconversion, ALT normalization, and overall virological and biochemical responses compared with placebo. Moreover, multivariate analysis determined the vaccine dose, patient age, and HBeAg baseline levels to be independent predictors of response to therapy. Of particular importance, the open-label extension demonstrated that prolonged treatment with this nanovaccine was also of benefit to initial non-responders, further raising seroconversion rates without proof of serologic relapse. The vaccine was tolerable with only mild-to-moderate side effects.^27^ These findings demonstrate the clinical potential of HBsAg-based nanovaccines like εPA-44 as a novel, safe, and efficient immunotherapeutic strategy for chronic hepatitis B with long-term serologic and virologic responses.

## Limitations and Future Directions

 While such promising outcomes have been discovered, numerous challenges remain. Most prominent is likely a dearth of animal models that better model HBV’s chronic, immune-tolerant natural history in humans. Most used HBV-transgenic or hydrodynamic injection models were poor simulations of ongoing infection and immune fatigue. Improved options, e.g., humanized mouse models that maintain HBV replication and immunopathogenesis, could better offer physiologically relevant models to validate the efficacy of nanovaccines.

 In addition, while nanoparticle platforms exhibit strong preclinical efficacy, clinical translation will require evaluation of the scalability of production, long-term safety, dosing regimens, and combination strategies (e.g., with immune checkpoint inhibitors or siRNA-based antivirals).

## Conclusion

 Overall, both protein-based and mRNA-based HBsAg nanovaccines demonstrated promising therapeutic potential for chronic hepatitis B treatment. Nanoplatforms such as HBsAg-loaded PLGA NPs, Ferritin NP–preS1 vaccines, DOTAP-assisted CLAN CpG nanoparticles + rHBsAg, NSG + NCG co-administered nanovaccine, and PP-SG nanovaccines all demonstrated potent immunogenicity, significant reduction of viral antigens and DNA, and strong T-cell activation with minimal hepatotoxicity in preclinical studies. Among them, targeting the PreS1 domain, such as in Ferritin NP–preS1 and PreS1-HBsAg mRNA-LNP vaccines, was an extremely effective means of inducing neutralizing antibody responses and eliciting rapid seroclearance. The εPA-44 liposome nanovaccine has shown significant clinical benefits, including higher rates of HBeAg seroconversion, ALT normalization, and composite virologic and biochemical response, with a good safety profile. Such findings are reflective of the potential of nanotechnology-based therapy in overcoming the constraints of conventional therapies, opening up functional cure approaches. Standardization and institutionalization of HBsAg-based nanovaccine clinical use in CHB elimination would need large-scale clinical trials, long-term follow-up studies, and vaccine reformulation.

## Competing Interests

 The authors declare no conflict of interest.

## Consent for Publication

 The authors declare no conflict of interest.

## Data Availability Statement

 Data sharing not applicable to this article as no datasets were generated or analysed during the current study.

## Ethical Approval

 Ethics approval was received from the ethics committee of Iran University of Medical Sciences (No. IR.IUMS.REC.1404.015).
